# Catheter Ablation of Atrial Fibrillation in a Patient With a Left Atrial Band Using Intracardiac Echocardiography (ICE)‑Guided 3D Geometry and a Visualizable Sheath: A Case Report

**DOI:** 10.7759/cureus.101148

**Published:** 2026-01-09

**Authors:** Shiori Watanabe, Saki Shinada, Yoshinori Shimooka, Yuichiro Kawamura, Naoki Nakagawa

**Affiliations:** 1 Department of Internal Medicine, Asahikawa Medical University, Asahikawa, JPN; 2 Department of Cardiology, National Hospital Organization Hokkaido Medical Center, Sapporo, JPN

**Keywords:** atrial fibrillation, intracardiac echo, left atrial band, radio-frequency ablation, steerable sheath

## Abstract

The left atrial band is a rare congenital structural anomaly in clinical practice. Its clinical significance in supraventricular arrhythmia and safe therapeutic strategies remains unclear. In this report, we present the case of a 68-year-old man with a history of myocardial infarction caused by thromboembolism secondary to atrial fibrillation who was admitted to our hospital for catheter ablation. Preprocedural contrast-enhanced CT revealed a cord-like structure, less than 2 mm in diameter, extending from the fossa ovalis to the left atrial roof. Catheter ablation was performed using the CARTO system with intracardiac echocardiography (CARTO SOUND) to create 3D geometry and visualize the sheath manipulation using a visualizable steerable sheath (VIZIGO) to confirm the spatial relationship between the steerable sheath and the left atrial band during manipulation of the ablation catheter. Post-procedural intracardiac echocardiography confirmed the integrity of the left atrial band. In conclusion, this case demonstrates that recognition of a left atrial band and the use of intracardiac echocardiography‑guided visualization with a visualizable sheath may assist in safe ablation procedures.

## Introduction

The left atrial band is a congenital structural anomaly found in approximately 2% of autopsy cases [1]; however, it is rarely detected in clinical practice, because the atrial band is often less than 2 mm thick and is frequently missed on transesophageal echocardiography (TEE). A left atrial band, composed of fibromuscular tissue, connects the left atrial side of the fossa ovalis to other areas of the left atrium [1]. An additional association between the left atrial band and the presence of a patent foramen ovale and Chiari network has been reported [2]. From a developmental perspective, the abnormal septum is likely part of the primitive septum that has shifted to the left, forming an additional compartment within the left atrium [3]. Left atrial band does not cause any characteristic clinical symptoms, but it has been reported to be associated with arrhythmias, cerebral infarction, and valvular heart disease. An increased incidence of premature atrial complexes has also been observed. Another study reported an association between left atrial bands and cardiogenic stroke [4] as well as mitral regurgitation [5]; however, their clinical significance and association with supraventricular arrhythmia remain unclear.

In cases undergoing catheter ablation, the presence of an atrial band may pose risks of catheter entrapment, thrombus formation, and treatment failure. To safely perform catheter ablation while avoiding damage to the left atrial band, it is essential to simultaneously visualize the position of the left atrial band and the position of the ablation catheter or steerable sheath being manipulated by the operator. This report aims to demonstrate how intracardiac echocardiography (ICE)‑guided 3D geometry and a visualizable sheath can assist in safe ablation in the presence of a left atrial band.

## Case presentation

**Figure 1:**
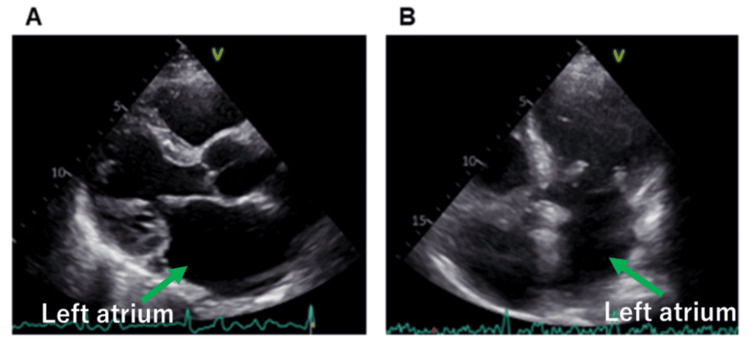
Transthoracic echocardiography revealed no abnormal structures in the left atrium.

**Figure 2:**
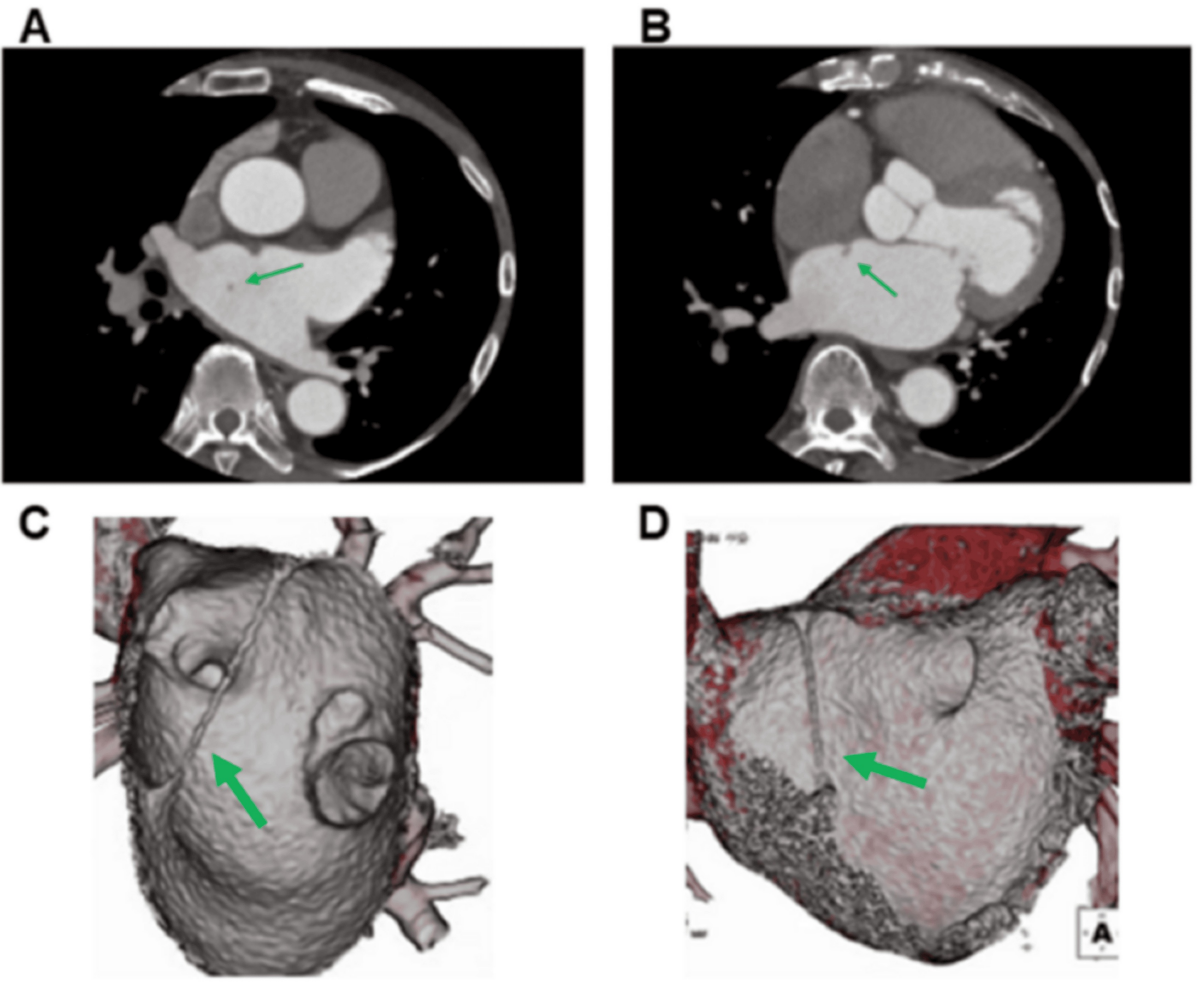
A cord-like structure connecting the fossa ovalis to the left atrial roof was observed on contrast-enhanced CT (A, B) and reconstructed 3D CT image of the inner (C) and anterior view (D). The green arrow indicates the atrial band.

A 68-year-old man with a history of myocardial infarction caused by thromboembolism secondary to persistent atrial fibrillation (AF) was admitted to our hospital for catheter ablation. A preprocedural transthoracic echocardiogram showed no abnormal structures (Figure 1); however, contrast-enhanced CT scans revealed a cord-like structure. The band measured 1.5-2 mm in diameter and approximately 44 mm in length, and it extended from the fossa ovalis to the left atrial roof (Figure 2). The position of the band poses a risk of interference with catheter manipulation, making it crucial to understand its spatial relationship with the ablation catheter to avoid damage during the procedure. Contrast-enhanced CT revealed that the space was wider posterior than anterior to the band (Figure 2C). When isolating the right pulmonary vein, damage to the band can be avoided by positioning the ablation catheter consistently to the right of the band. Similarly, when isolating the left pulmonary vein, damage can be avoided by positioning the ablation catheter consistently posterior to the band.

**Figure 3:**
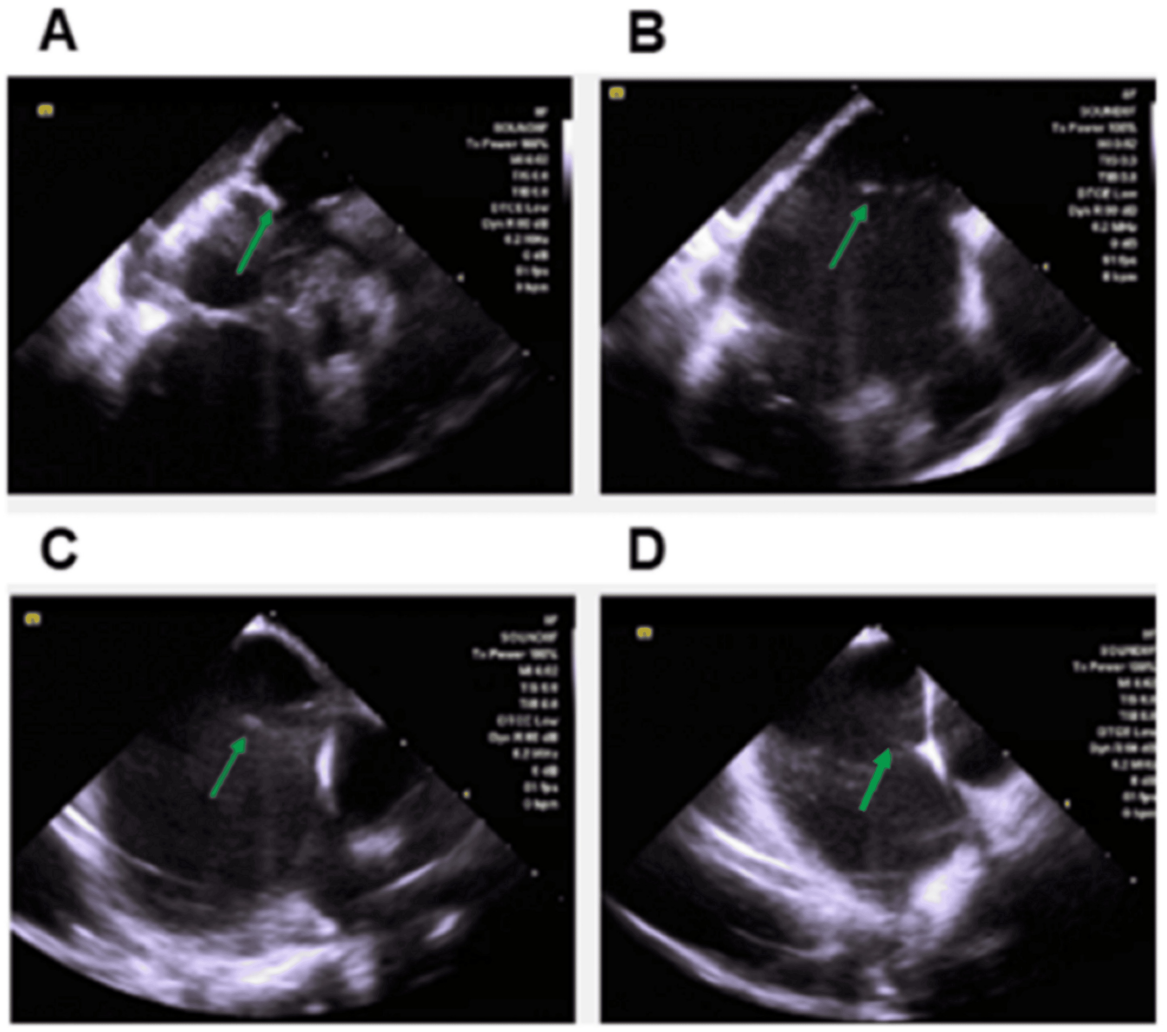
A 3D geometry using CARTO SOUND to trace the left atrial band. The green arrow indicates the atrial band.

**Figure 4:**
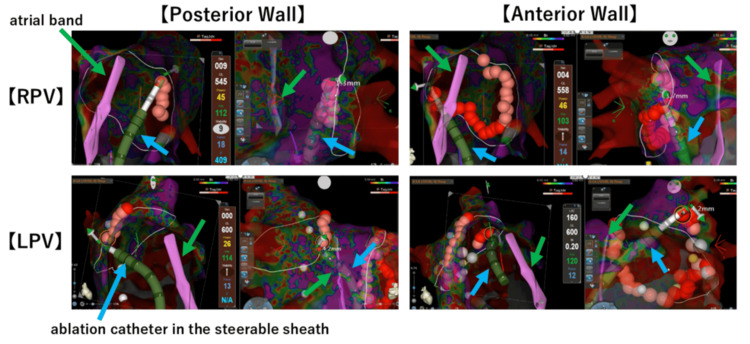
Bilateral pulmonary vein isolation is performed by manipulating the catheter and confirming the relationship between the 3D geometry of the left atrial band, as created with CARTO SOUND, and the position of the VIZIGO sheath, on two screens. The green arrow indicates the atrial band. The blue arrow indicates the ablation catheter and the steerable sheath.

Catheter ablation was performed using an electro-anatomical mapping system (CARTO; Biosense Webster, Inc., Irving, CA). We continuously visualized the left atrial band and the entire left atrium using an ICE catheter (SoundStar, Biosense Webster, Inc.) (Figure 3) and created a 3D geometry using CARTO SOUND. The band showed almost no mobility, and even with ICE applied, no significant movement was observed except for respiratory motion. For atrial septal puncture, we targeted a location on the right side, slightly lower than the atrial septal attachment point of the band, using ICE guidance. Using 3D geometry as a reference, we mapped the left atrium with an OCTARAY mapping catheter (Biosense Webster, Inc.) in the AF rhythm. To avoid damage to the left atrial band during ablation, clearly identifying the positional relationship between the ablation catheter and the left atrial band was necessary. Therefore, we inserted a contact-force catheter (ThermoCool SmartTouch; Biosense Webster, Inc.) into a visualizable steerable sheath (VIZIGO; Biosense Webster, Inc.) and manipulated it under visualization using the left atrial band from two perpendicular screens (Figure 4). In this case, the total radiofrequency ablation time was 24 minutes, the average contact force was 10-25g, the power was 45W, and the ablation index was 400 in the posterior wall and 450 in all other areas. The fluoroscopy time was 11 minutes, and the total procedural duration was 175 minutes.

**Figure 5:**
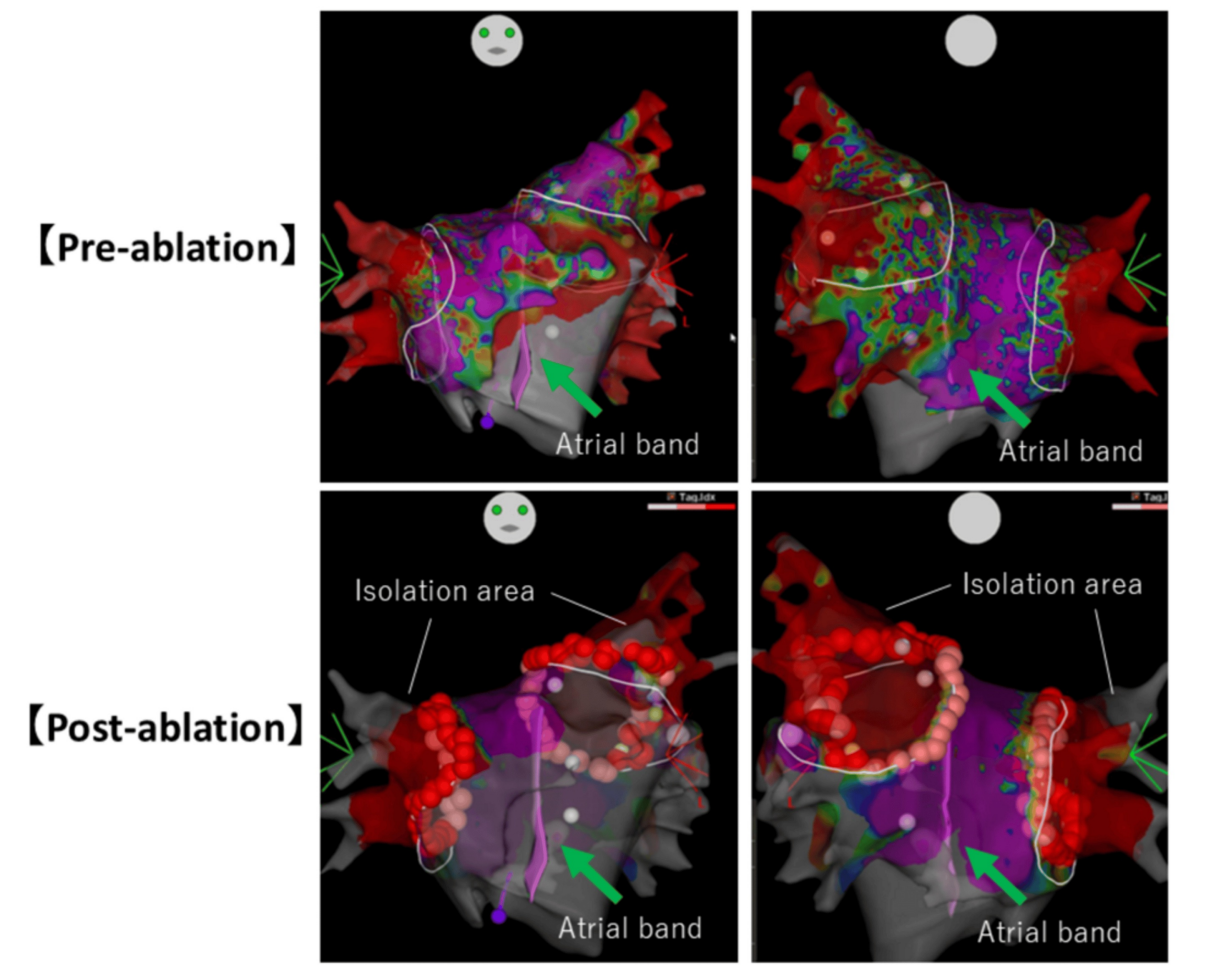
Voltage mapping of the pre-ablation and post-ablation. The green arrow indicates the atrial band.

In our case, the left atrial band was attached to the left atrial roof via the atrial septum. As we did not anticipate the left atrial band to be the source of the arrhythmia, our plan was limited to performing pulmonary vein isolation; therefore, we did not perform ablation of the structure. We successfully performed bilateral pulmonary vein isolation, as confirmed by voltage mapping (Figure 5). The patient had no complications during or after the procedure. We clarified that the patient underwent regular outpatient visits for two years. No recurrence of AF occurred during this time.

## Discussion

This case highlights the importance of careful preprocedural recognition and intra-procedural visualization of the left atrial band in patients with AF.

The left atrial band can often be overlooked in transesophageal echocardiography due to variations in its location, thickness, and length, as well as a general lack of awareness among clinicians. Several case reports have suggested that contrast-enhanced CT is an effective modality for detecting the left atrial band. Left atrial band has been linked to complications, such as mitral regurgitation, when attached to the mitral valve [5]. Additionally, they may be discovered after a stroke. As a cause of cerebral infarction, studies have reported that the presence of a cord-like structure alone may lead to thrombosis, and that cases with an atrial band are often associated with a patent foramen ovale (PFO) [1,4]. In cases where a left atrial band is detected, it is advisable to investigate for PFO. Additionally, although no reported cases were found, performing ablation in cases with a left atrial band carries the risk of entrapment or damaging the band itself, potentially causing intraoperative embolism or cardiac tamponade.

The ICE catheter is widely used during ablation procedures, primarily for transseptal puncture and CT merging of the left atrium, ventricle, and aortic cusps. Additionally, an ICE catheter is useful for delineating abnormal structures in the left atrium, such as the left atrial band. It also allows visual confirmation of the contact between the structure and the ablation catheter. To prevent inadvertent damage, we used ICE (CARTO SOUND) to create a 3D anatomical model and a steerable sheath that could be visualized (VIZIGO) to confirm the spatial relationship between the ablation catheter and the left atrial band. Pulmonary vein isolation was successfully achieved without complications, and the left atrial band remained intact.

Pathologically, the left atrial band consisted of fibrous and muscle tissues with normal myocardial fibers without Purkinje cells [5]. Reports on the electrical conduction of the left atrial band are rare; however, two case reports have described its electrophysiological properties. One involved a cord-like structure extending from the atrial septum to the posterior wall of the left atrium. During posterior wall isolation, residual local potentials were observed at the posterior wall attachment site, which disappeared after ablation of the atrial septal attachment site [6]. Another report described a cord-like structure connecting the atrial septum to the posterior wall of the left atrium. An electrode catheter was placed on the cord-like structure, and pacing from both the septal and posterior walls demonstrated conduction across the sides [7]. Based on the above findings, clinicians should exercise caution in cases where the left atrial band is present within the ablation field, as this may result in incomplete block lines. Thus, ablation of the left atrial band may be necessary when it is capable of inducing arrhythmias or when it is included within the planned ablation area. In our case, the left atrial band was attached to the left atrial roof via the atrial septum. As this was the first treatment case, we planned to perform only pulmonary vein isolation; therefore, the left atrial band was not included in the treatment area, and the left atrial band ablation was not performed. Instead, our strategy focused on visualizing the procedure to avoid damage to the structure.

This case report has several limitations. First, cases with a left atrial band are rare, so this report is a single case. Second, no direct electrophysiological study has been performed on the left atrial band. To resolve these challenges, we need to accumulate similar cases. This case has been followed up in the outpatient setting for two years to date. However, further long-term follow-up is necessary to assess the potential for recurrence of the left atrial band or its role in causing other arrhythmias, as well as its potential to cause embolism.

## Conclusions

The presence of a left atrial band can increase technical difficulties during catheter ablation for AF due to anatomical challenges. Therefore, awareness of their presence before the catheter is important to establish a safe and effective treatment strategy. For cases with abnormal structures in the left atrium, visualization with CARTO SOUND and VIZIGO sheaths is useful to avoid iatrogenic injury to these structures.
